# Effects of Short-Term Continuous Subcutaneous Insulin Infusion on Fasting Plasma Fibroblast Growth Factor-21 Levels in Patients with Newly Diagnosed Type 2 Diabetes Mellitus

**DOI:** 10.1371/journal.pone.0026359

**Published:** 2011-10-26

**Authors:** Mengliu Yang, Jing Dong, Hua Liu, Ling Li, Gangyi Yang

**Affiliations:** 1 Department of Endocrinology, the Second Affiliated Hospital, Chongqing Medical University, Chongqing, China; 2 Department of Pediatrics, University of Mississippi Medical Center, Jackson, Mississippi, United States of America; Genentech Inc., United States of America

## Abstract

**Background:**

To investigate the effects of short-term continuous subcutaneous insulin infusion (CSII) on plasma fibroblast growth factor-21 (FGF-21) levels in patients with newly diagnosed type 2 diabetes mellitus (nT2DM).

**Method:**

Sixty-eight patients with nT2DM (nT2DM group), and 52 gender-, age- and body mass index (BMI) -matched normal glucose tolerance (NGT group) controls participated in the study. 30 nT2DM patients with FBG≥14.0 mmol/L were treated with CSII for 2 weeks, and were underwent a euglycemic–hyperinsulinemic clamp pre- and post-treatment. Plasma FGF-21 concentrations were measured with a commercial ELISA kit. The relationship between plasma FGF-21 levels and metabolic parameters was also analyzed.

**Results:**

Fasting plasma FGF-21 levels were higher in the nT2DM group than in NGT groups (1.60±0.08 vs. 1.13±0.26 µg/L, *P*<0.01). In nT2DM patients, fasting plasma FGF-21 concentrations were significantly decreased after CSII treatment for 2 weeks (1.60±0.08 vs.1.30±0.05 µg/L, *P*<0.05), accompanied by a significant increase in the whole body glucose uptake (M value) and blood glucose control. The changes in plasma FGF-21 levels (ΔFGF-21) were positively associated with the amelioration of insulin resistance shown by the changes in M value.

**Conclusion:**

Plasma FGF-21 level is associated with whole body insulin sensitivity and significantly reduced following short-term CSII treatment.

## Introduction

Fibroblast growth factors (FGFs) represent a group of peptides that regulate diverse biological functions, including cell differentiation, cell growth, and angiogenesis [1], [2]. Recently, a subfamily of FGFs that interact with nuclear receptors has been identified that plays an important role in liver, bone, and adipose tissue metabolism [3]–[4]. In murine models, Fibroblast growth factor 21 (FGF-21) was reported to be expressed predominantly in liver [5], but its expression has also been reported in adipose tissue and pancreatic β-cells [6]. It is a potent metabolic regulator shown to improve insulin resistance as well as to reduce overall body weight and adipose mass [7]–[13]. Pharmacologically, FGF-21 induced glucose uptake through the induction of glucose transporter-1 (GLUT1) in adipocytes, and *in vivo* treatment with FGF-21 resulted in amelioration of glucose and lipid metabolism in both murine and nonhuman primate models of diabetes or obesity [11], [14]–[15]. Furthermore, mice overexpressing FGF-21 was resistant to diet-induced obesity and exhibited improved glucose homeostasis [11]. FGF-21 knockout (FGF-21KO) mice developed mild obesity and impaired glucose homeostasis, as these mice aged [16]. We had previously demonstrated that plasma FGF-21 levels were elevated in patients with type 2 diabetes mellitus (T2DM) [17], and were decreased in response to treatment with rosiglitazone [18]. More recently, Zhang et al. [19] found that FGF-21 concentrations were elevated in obese nondiabetic individuals compared with lean healthy control subjects and that the circulating levels correlated positively with adiposity and fasting insulin and negatively with HDL cholesterol. Conversely, in patients with anorexia nervosa, plasma FGF-21 concentrations were decreased and increased following weight gain [20].

Since the role of FGF-21 in human physiology remains to be elucidated, it is important to characterize the effect of common anti-diabetic treatment on the plasma levels of FGF-21. Transient insulin treatment optimized by continuous subcutaneous insulin infusion (CSII) is effective in rapidly reducing hyperglycemia and glucotoxicity, and leads to the restoration of insulin sensitivity in T2DM [21]. CSII, which mimics physiological insulin secretion, becomes feasible, and results in fewer side effects by flexible and episodic insulin adjustment. Early intensive insulin therapy in patients with newly diagnosed T2DM (nT2DM) recovers β-cell function to a certain degree, along with a concurrent improvement in insulin sensitivity [22], [23]. Thus, the aim of this study was to examine plasma FGF-21 levels in nT2DM patients and to evaluate the effects of CSII treatment for two weeks on plasma FGF-21 level in these patients.

## Results

### The clinical characteristics and fasting plasma FGF-21 levels

The clinical characteristics of the two groups did not show significant difference in gender distribution, age, BMI, Fat %, blood pressure and HDL-C (Table 1). WHR, TC, TG, LDL-C, FBG, 2 h OGTT and HbA1c in nT2DM group were higher than NGT group (*P*<0.05 and *P*<0.01). Compared with the NGT group, the patients with nT2DM had higher HOMA-IR (*P*<0.01), but lower fasting plasma insulin (FINS) and HOMA-IS (*P*<0.01). There was significant difference in fasting plasma FGF-21 levels between the nT2DM and NGT groups (1.60±0.08 vs.1.13±0.26 µg/L, *P*<0.01). However, no sexual dimorphism became apparent in plasma FGF-21 levels (*P* = NS).

**Table 1:** Clinical characteristics and FGF-21 levels of study subjects.

Group	T2DM	Post-treatment	NGT
BMI(kg/m2)	24.0±2.3	24.5±2.3	23.9±4.2
WHR	0.89±0.04*	0.89±0.05	0.85±0.08
Body fat(%)	27.7±2.6	27.7±2.6	30.0±7.9
SBP(mmHg)	130±11	123±13	125±18
DBP(mmHg)	81±8	75±5	80±9
TG(mmol/L)	3.74±1.98**	3.21±1.51▴	1.79±1.24
TC(mmol/L)	5.07±0.83*	3.37±2.17▴▴	4.71±2.02
HDL-C (mmol/L)	1.32±0.93	1.19±0.25	1.31±0.36
LDL-C (mmol/L)	3.39±1.14**	2.56±0.71▴▴	2.69±0.68
FFA (umol/L)	0.95 (0.54–2.55)	0.81 (0.12–2.86)	0.85±0.53
HbA1c(%)	10.7±1.5**	8.98±1.16▴▴	5.9±0.3
FBG(mmol/L)	14.5±1.6**	6.0±0.9▴▴	5.7±0.3
2hOGTT(mmol/L)	21.6±4.6**	7.1±0.7▴▴	6.0±0.9
FINS(mU/L)	6.7 (2.59–10.37)*	8.81 (4.61–26.56)▴	12.37(5.29–60.20)
PINS(mU/L)	20.65 (4.1–56.26)	45.45(12.44–92.78)▴	29.09(7.09–87.40)
HOMA-IR	4.28 (1.7–6.47)**	2.3 (1.09–7.2)▴▴	2.98(0.71–15.41)
HOMA-IS	13.20 (4.09–21.8)**	78.21(46.75–204.31)▴▴	140.92(32.5–539.2)
FGF-21(µg/L)	1.60±0.08**	1.30±0.05▴	1.13±0.26
M (mg/kg/min)	2.99±0.42	5.10±0.51▴▴	-------------
M/I[mg/kg/Min/(mU/l)]	0.19±0.04	0.43±0.09▴▴	--------------

Data are means ± S.D. or median. BMI, body mass index; SBP, systolic blood pressure; DBP, diastolic blood pressure; WHR, waist-to-hip ratio; fat%, visceral fat %; FBG, fasting blood glucose; 2 h OGTT, 2 h post-glucose load blood glucose; FINS, fasting plasma insulin; PINS, 2 h plasma insulin after glucose overload; HOMA-IR, HOMA-insulin resistance index; HOMA- IS, HOMA - β-cell insulin secretion index; FFA, free fatty acids; TG, total triglyeride; TC, total cholesterol; HDL-C, high-density lipoprotein cholesterol; LDL-C, low-density lipoprotein cholesterol; HbA1c,glycosylated hemoglobin.

*P<0.05,

**P<0.01 compared with NGT group;

▴ *P*<0.05,

▴▴ *P*<0.01 compared with pre-treatment.

### Relationship between fasting plasma FGF-21 levels and metabolic parameters

Bivariate correlation analyses were performed to assess relationships between plasma FGF-21 concentrations and body composition or metabolic parameters. Fasting plasma FGF-21 was found to correlate positively and significantly with HbA1c (*r* = 0.59, *P*<0.01). systolic blood pressure (SBP, *r* = 0.17, *P*<0.05), diastolic blood pressure (DBP, *r* = 0.18, *P*<0.05), FBG (*r* = 0.52, *P*<0.01), 2 h OGGT (*r* = 0.57, *P*<0.01), HDL-C (*r* = 0.18, *P*<0.05) and FFA (*r* = 0.18, *P*<0.05), but negatively with FINS (*r* = −0.28, *P*<0.01), PINS (*r* = −0.24, *P*<0.01) and HOMA-IS (*r* = −0.54, *P*<0.01). Multiple regression analysis showed that DBP, WHR, 2 h plasma insulin after glucose overload (PINS), 2 h OGTT and FFA were independent related factors influencing plasma FGF-21 levels (Y_FGF-21_ = 3.186+0.058X_2 h OGTT_−0.007X_PINS_+0.491X_FFA_+3.206X_WHR_+0.021X_DBP_).

### The effects of CSII on clinical characteristics and FGF-21 levels in nT2DM patients

**Figure 1:**
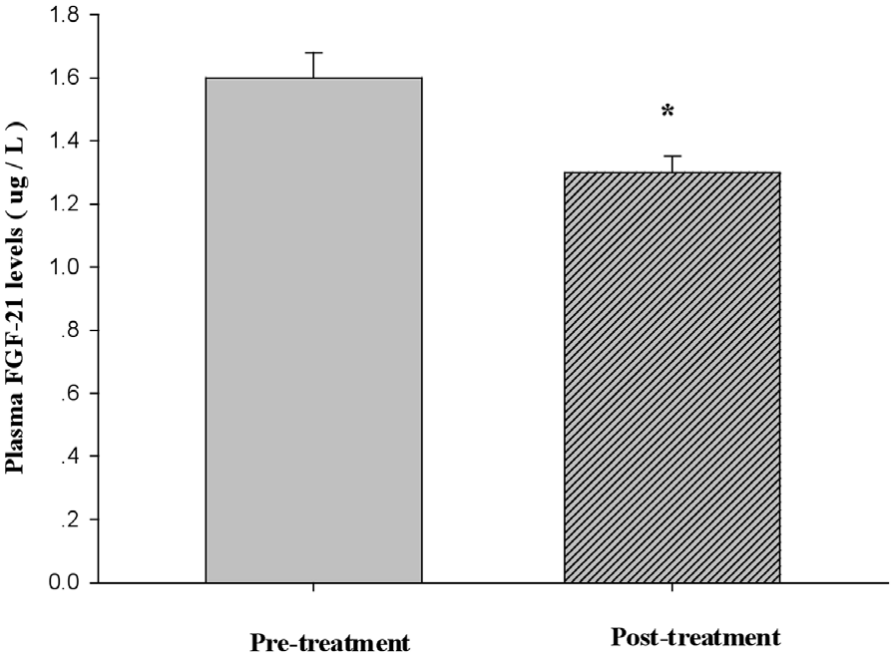
Plasma FGF-21 levels pre- and post-treatment with CSII in 30 nT2DM patients with FBG≥14.0 mmol/L. * *P*<0.05 vs. pre-treatment.

30 nT2DM patients treated with CSII achieved excellent glycemic control within 3–5 days and maintained euglycemia in the following 2 weeks. After 2 week of CSII therapy, blood glucose control was remarkably improved. As shown in Table 1, there was a significant decrease in FBG, 2 h OGTT and HbA1c (all *P*<0.01), and an increase in insulin levels, including FINS and PINS (*P*<0.05 and *P*<0.01). Treatment with CSII decreased plasma TG, TC and LDL-C levels (*P*<0.05 or *P*<0.01). Also, elevated fasting plasma FGF-21 levels were decreased (1.60±0.08 vs. 1.30±0.05, *P*<0.05, Figure 1) concomitant with ameliorations in insulin resistance and β-cell dysfunction, which were shown by a significant decrease in HOMA-IR values and a significant increase in HOMA-IS values (both *P*<0.01, Table 1).

To directly examine the quantitative effect of CSII treatment on insulin sensitivity, we performed EHC on the same group of patients pre- and post-treatment. During the last hour of the clamps, glucose concentrations were 5.9±0.3 and 6.0±0.4 mmol/L, respectively, pre and post CSII treatment (differences not statistically significant). Fasting insulin concentrations were 6.7 (2.59–10.37) mU/L pre CSII treatment and increased to 8.81 (4.61–26.56) mU/L (*P*<0.05). During the last hour of the clamps, serum insulin concentrations were 28.4±4.9 and 30.2±5.9 mU/L pre- and post- CSII treatment, respectively (differences not significant). CSII treatment resulted in a significant increase in glucose disposal rate shown as M values (5.10±0.51 vs. 2.99±0.42 mg/kg/min, *P*<0.01) and insulin sensitivity index shown as M/I (0.43±0.09 vs. 0.19±0.04 mg/kg/min mU/l, *P*<0.01, Table 1). We also assessed the associations between the changes of plasma FGF-21 (ΔFGF-21) and several parameters pre- and post- CSII treatment. Interestingly, ΔFGF-21 was positively associated with ΔDBP (*r* = 0.478, *P*<0.01), but negatively ΔHDL (*r* = −0.395, *P*<0.01). More important, to investigate the association of plasma FGF-21 and insulin resistance, multiple regression analyses in the subgroup of patients with EHC revealed a strong association ΔFGF-21 with M values (R2 = 0.565, B = −0.266, *P*<0.01).

## Discussion

FGF-21 was discovered during a high-throughput assay for secreted proteins that increased glucose uptake in 3T3L-1 adipocytes [11]. Subsequent studies [4], [11], [15] showed that administration of recombinant FGF-21 in rodent models of diabetes and in diabetic rhesus monkeys improved blood glucose and the lipid profile. In addition, it had been demonstrated that plasma FGF-21 levels were elevated in insulin-resistant states (obesity, IGT/IFG, T2DM) and were inversely correlated with both peripheral and hepatic insulin sensitivity [24]. However, fewer studies compared plasma FGF-21 levels before and after anti-diabetic agents. In the current study, we found that plasma FGF-21 levels were elevated in patients with new-onset T2DM compared with the gender-, BMI- and age-matched NGT subjects. Furthermore, we found that fasting plasma FGF-21 was positively and significantly correlated with SBP, DBP, FBG, 2 h OGTT, HbA1c, HDL-C and FFA, but negatively with FINS, PINS and HOMA-IS. Therefore, the most likely explanation for these findings is that elevated FGF-21 levels in nT2DM patients might represent a compensatory mechanism in response to decreased insulin sensitivity or impairment of glucose metabolism. Another explanation could be that elevated FGF-21 levels in these patients may be a compensatory response to decreased fasting plasma insulin levels due to a defect in beta cell function. These results were consistent with our and other reports [17], [19] in humans that demonstrated elevated plasma FGF-21 concentration in obesity, IGT, and T2DM. These data further suggested that T2DM or obesity might also have an FGF21-resistant state [25]. We had previously demonstrated a decrease in fasting plasma FGF-21 after treatment with rosiglitazone in T2DM [18]. We considered that this decrease could be caused by the improved insulin resistance via rosiglitazone treatment. In the current study, blood glucose, both postprandial and fasting glucose levels, were nearly normalized within 7 days and the euglycemia was maintained for 2 weeks by CSII therapy in patients with severe nT2DM. Meanwhile, TG, TC, LDL-C and HbA1c were also significantly lower after CSII therapy. These findings indicated the amelioration of glucose-lipid metabolism. In addition, increasing insulin levels and HOMA-IS indicated an amelioration of glucotoxicity and β-cell dysfunction after treatment with CSII in these patients. Furthermore, EHC showed that M and M/I values were significantly increased by CSII therapy, suggesting an amelioration of insulin resistance. Interestingly, along with the rapid improvement in glucose-lipid metabolism and insulin resistance, and elevated plasma insulin, we observed a dramatic decrease in fasting plasma FGF-21 concentrations in these patients. More importantly, the reduction of plasma FGF-21 levels was found to associate with the amelioration of insulin resistance shown by a significant relationship between ΔFGF-21 and M values in T2DM patients with EHC. Hence, the regulation of circulating FGF-21 might be influenced by metabolic control, insulin resistance and β cell function. Decreased circulating FGF-21 after CSII treatment might also mediate the improvement of insulin resistance in the nT2DM patients. However, we cannot also rule out the possibility that infusion of insulin is reducing FGF-21 levels. In addition, CSII treatment may improve FGF-21 action and consequently alleviate FGF-21 resistance, and reduces plasma FGF-21 levels, but this needs further investigation.

In conclusion, our study found that circulating FGF-21 levels were significantly increased and significantly associated with HbA1c, FBG, 2 h OGTT, HDL-C, FFA, insulin and HOMA-IS in nT2DM. More importantly, we presented, for the first time to our knowledge, a novel data that CSII treatment significantly decreased plasma FGF-21 levels in these patients. The reduction of plasma FGF-21 levels was found to associate with the amelioration of insulin resistance shown by a significant relationship between ΔFGF-21 and M values in EHC. Taking these findings into consideration, our results supported the hypothesis that FGF-21 may be an insulin sensitizing cytokine in humans.

## Materials and Methods

### Subjects

120 volunteers were involved in this study and categorized into two groups. Sixty-eight subjects with nT2DM and 52 control subjects participated in the study. The diagnostic criteria of T2DM was based on a 75 g oral glucose tolerance test (OGTT) recommended by World Health Organization criteria [26]. These patients had not taken any diabetic medications/diet prior to the present study. Patients with a history of unstable proliferative retinopathy, evidence of liver disease, renal dysfunction, severe cardiac problems, uncontrolled hypertension, or type 1 diabetes were excluded. 52 healthy volunteers, age- and BMI matched with nT2DM groups, were chosen as the normal controls (NGT group). These subjects without clinical evidence of major disease were recruited from an unselected population that underwent routine medical check-ups and did not receive any therapy. Their non-diabetic status was confirmed with a normal OGTT. This study was carried out in accordance with the recommendations of the Declaration of Helsinki. The study was approved by the Human Research Ethics Committee of Chongqing Medical University. A written informed consent was obtained from all participants in this study.

### Study design

Anthropometric measurement was performed in the morning, before breakfast, with the subject wearing light clothing, without shoes. Body weight and height were measured by the same observer using a scale and a wall-mounted stadiometer to 0.5 kg and 0.5 cm, respectively. Waist and hip circumferences were measured, and the waist to hip ratio (WHR), which provided valuable information about the distribution of body fat, was calculated as the ratio of waist and hip circumferences. Standard procedures were followed [26], and the average of the two measurements was taken. After 1.75 g/kg body weight (maximum 75 g) of glucose was administered as 20% glucose solution, venous blood was sampled at baseline and 120 min. Plasma glucose level and HbA1c were immediately measured by the glucose-oxidase technique or anion exchange HPLC respectively, to avoid anaerobic glucose consumption. Plasma was then stored at −80°C for determination of plasma free fatty acid (FFA), insulin, FGF-21 and blood fat levels.

Thirty patients only with FBG≥14.0 mmol/L were admitted to the hospital, and then treated with intensive insulin therapy by CSII with MiniMed 712E insulin pumps (Medtronic MiniMed, Northridge, CA, USA). The initial daily insulin (Insulin aspart, Novo Nordisk, Denmark) dose was calculated as follows: total insulin dose daily = (0.4–0.6) unit ×body weight (kg). The basal rate (Units/hour) was calculated as 50% of the total insulin dose, and other 50% was administered as preprandial boluses before each of three meals. The basal and boluses of insulin infusion were adjusted according to preprandial and postprandial capillary blood glucose. Excellent blood glucose control was defined as FBG<6.1 mmol/l and postprandial blood glucose (2hPBG)<7.8 mmol/l, and was achieved within 3–5 days. After 2 weeks of CSII, insulin treatment was stopped. Other 38 patients with FBG≤14.0 mmol/L were treated with anti-diabetic agents or only diet and exercise.

Euglycemic–hyperinsulinemic clamp (EHC) was performed in 16 of the 30 T2DM subjects before CSII, and was repeated at least 24 h after insulin cessation. Briefly, after an overnight fast, an intravenous catheter was placed in the antecubital vein to infuse insulin and glucose. Another catheter was placed retrograde in the dorsal vein of the contralateral hand for blood withdrawal. This arm was wrapped with a heating blanket (∼70°C) to arterialize venous blood. Regular human insulin (Humulin; Eli Lilly, Indianapolis, IN) was infused at a rate of 2 mU · kg^−1^· min^−1^ to raise insulin concentrations to 200 µU/ml and a variable infusion of 20% glucose was administered to maintain plasma glucose at ∼6 mmol/L for 2 h. During the procedure, plasma glucose was measured every 5 min to guide the glucose infusion. The rate of glucose disposal was defined as the glucose infusion rate (GIR) during the stable period of the clamp and was related to body weight (M value, mg/kg per min) [27]. The insulin sensitivity index (M/I) was calculated as the whole body glucose uptake (M value) divided by insulin concentration (I value). No anti-diabetic agents, oral contraceptives or anti- hyperlipidemic agents were used during the study. No subject dropped out of the study. Blood samples were taken before CSII treatment and after at least 24 h insulin cessation for the measurements of metabolic parameters and plasma FGF-21 levels.

### Plasma biochemical parameters and FGF-21

Plasma FGF-21 levels were measured with a commercial ELISA kit (Adipobiotech, Inc., Beijing, China). The linear range of the assay was 0.5–8.5 µg/L, and the standard range was 0.13–20 µg/L. The inter- and intra-assay coefficients of variation were 10 and 5% respectively. Plasma insulin concentration was measured by radioimmunoassay (Diagnostic Products, Los Angeles, CA). FFA were measured with a commercial assay kit (Randox Laboratories Ltd, Antrim, UK). Triglyceride (TG), cholesterol (TC), high-density lipoprotein cholesterol (HDL-C) and lowdensity lipoprotein cholesterol (LDL-C) concentrations were determined enzymatically. Percent body fat (Fat %) was determined by bioelectrical impedance analysis (Tanita, Inc., Tokyo, Japan). The homeostasis model assessment of IR (HOMA-IR) and the HOMA of β-cell insulin secretion (HOMA-IS) were calculated from fasting insulin (FINS) and glucose levels using following equations:
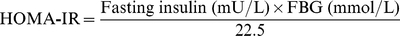


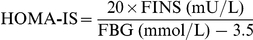

[28].

### Statistical analysis

Statistical analyses were performed using the SPSS 15.0 software (SPSS, Chicago, IL, USA). Since the distributions of plasma insulin, HOMA-IR and HOMR-IS values were skewed, logarithmically transformed values were used for statistical analysis. Baseline characteristics of case and control subjects were compared by *t*-test. The paired *t*-test was used to compare differences in biochemical characteristics and FGF-21 levels between pre- and post-treatment with CSII in nT2DM group. Bivariate correlation and multiple regression analyses were used to examine the association between fasting plasma FGF-21 levels and the values of other biomarkers. There was no existence of the multicollinearity between all the variables included in the multiple regression analyses. All of the statistical analyses were two-sided, and all data were presented as means ±S.D. with a *P* value<0.05 considered statistically significant.
